# FAIR Data Reuse in Traumatic Brain Injury: Exploring Inflammation and Age as Moderators of Recovery in the TRACK-TBI Pilot

**DOI:** 10.3389/fneur.2021.768735

**Published:** 2021-11-03

**Authors:** J. Russell Huie, Austin Chou, Abel Torres-Espin, Jessica L. Nielson, Esther L. Yuh, Raquel C. Gardner, Ramon Diaz-Arrastia, Geoff T. Manley, Adam R. Ferguson, The TRACK-TBI Investigators

**Affiliations:** ^1^Brain and Spinal Injury Center, Department of Neurosurgery, University of California, San Francisco, San Francisco, CA, United States; ^2^San Francisco Veterans Affairs Medical Center, San Francisco, CA, United States; ^3^Department of Psychiatry and Behavioral Sciences, University of Minnesota, Minneapolis, MN, United States; ^4^Institute for Health Informatics, University of Minnesota, Minneapolis, MN, United States; ^5^Department of Radiology, University of California, San Francisco, San Francisco, CA, United States; ^6^Department of Neurology, University of California, San Francisco, San Francisco, CA, United States; ^7^Department of Neurology, Perelman School of Medicine, University of Pennsylvania, Philadelphia, PA, United States

**Keywords:** leveraging data science for traumatic brain injury prevention, evidence based healthcare, precision medicine, data sharing, aging, inflammation, proteomics, outcomes

## Abstract

The guiding principle for data stewardship dictates that data be FAIR: findable, accessible, interoperable, and reusable. Data reuse allows researchers to probe data that may have been originally collected for other scientific purposes in order to gain novel insights. The current study reuses the Transforming Research and Clinical Knowledge for Traumatic Brain Injury (TRACK-TBI) Pilot dataset to build upon prior findings and ask new scientific questions. Specifically, we have previously used a multivariate analytics approach to multianalyte serum protein data from the TRACK-TBI Pilot dataset to show that an inflammatory ensemble of biomarkers can predict functional outcome at 3 and 6 months post-TBI. We and others have shown that there are quantitative and qualitative changes in inflammation that come with age, but little is known about how this interaction affects recovery from TBI. Here we replicate the prior proteomics findings with improved missing value analyses and non-linear principal component analysis and then expand upon this work to determine whether age moderates the effect of inflammation on recovery. We show that increased age correlates with worse functional recovery on the Glasgow Outcome Scale-Extended (GOS-E) as well as increased inflammatory signature. We then explore the interaction between age and inflammation on recovery, which suggests that inflammation has a more detrimental effect on recovery for older TBI patients.

## Introduction

As data science technologies rapidly advance, we are able to collect, store, and leverage more clinical traumatic brain injury (TBI) research data than ever before, opening new opportunities for precision medicine in neurotrauma. For example, machine learning has the potential to harness the full information contained within medical records to place individual patients into their own unique position in a multidimensional outcome space for TBI (1, 2). However, in order to realize the potential of biomedical “big-data,” there needs to be effective data management and governance that will enable responsible application of advanced analytics. In order to ensure that research data can be optimally utilized and shepherded appropriately, governance bodies have determined that biomedical data need to be made FAIR: findable, accessible, interoperable, and reusable (3). FAIR principles provide a roadmap for how data can be shared and reused in a way that is beneficial to the data originator, to the reuser, and ideally to the field as a whole. The end goal of FAIR data is to crowdsource secondary analyses, power machine-learning-based exploratory analyses, and accelerate novel hypothesis generation. In addition, data reuse encourages reproducibility and helps ensure that the scientific community extracts as much information as possible from costly federal investments in research dollars and data collection time (4–6). The Transforming Research and Clinical Knowledge in Traumatic Brain Injury (TRACK-TBI) study is an example of early adoption of large-scale FAIR data sharing with the goal of advancing knowledge discovery in clinical TBI (7–9). The initial TRACK-TBI Pilot project generated a dataset of 586 subjects from three level 1 trauma centers that were deeply phenotyped across 900+ variables spanning clinical variables, biofluid biomarkers, imaging biomarkers, and neuropsychiatric and cognitive outcomes. Primary analysis papers were purpose-curated around specific hypotheses, resulting in 38 papers (as of July 2021), and staged improvement in data curation quality over time. Ultimately, the multidimensional curation across variables began to reach sufficient maturity such that later versions of the dataset were primed for advanced multidimensional analytics, including application of machine learning and other artificial intelligence tools to predict outcomes at the level of individual subjects while taking into account ever larger numbers of features (10–12). In addition, the TRACK-TBI Pilot gave rise to an 18-center TRACK-TBI study that has generated 52 papers (and counting).

The goal of the current study is to demonstrate the real-world application of FAIR principles to TBI and expand on prior findings by combining and reusing TRACK-TBI Pilot data from two previously published studies (10, 11). Prior work demonstrated that a multianalyte blood biomarker panel correlates with TBI outcomes. Much of the variance in blood biomarkers reflected inflammatory pathways, and patients with higher pro-inflammatory expression were more likely to have been diagnosed by neuroradiologists as having at least one pathology on computed tomography scan (CT positive) and performed significantly worse on the California Verbal Learning Task and the Glasgow Outcome Scale-Extended (GOS-E) than those expressing a less inflammatory expression profile (10).

Here we revisit these data and cross-curate them with age demographics to determine the extent to which age moderates the role of inflammation on recovery after TBI. As we and others have shown in previous work, the body reacts differently to TBI depending on age (13). Patients with TBI over the age of 40 are more likely to decline over the first 5 years after injury, and those over 55 have an increased risk of developing dementia and Parkinson's disease (14–16). The patterns of expression for inflammatory patterns in particular are sensitive to age as has been in shown in both pre-clinical and clinical studies of TBI, spinal cord injury (SCI), and stroke (17–21). Despite this, little is known about the nature of the interaction between age and inflammation on outcome after TBI.

The current data reuse study aims to first determine the effect of age on outcome in the TRACK-TBI Pilot cohort. We then sought to replicate the multivariate analyses of the protein biomarker panel using improved techniques for handling missing data and non-linear dimensionality reduction while verifying that the biomarker profile is associated with functional outcome. Finally, we explore the extent to which age moderates the ensemble biomarker profile as a predictor of recovery after TBI.

## Methods

### Data Reuse

The current dataset was created by merging and cross-curating data from two previous subsets of the full TRACK-TBI Pilot dataset that were used previously (10, 11). This dataset was composed of TRACK-TBI participants (*N* = 586) aged 17+ years who were enrolled between April 2010 and January 2011 from three level 1 trauma centers in the United States. Assessment of evidence for acute TBI within 24 h of injury by non-contrast head CT was used as the primary inclusion criterion. Exclusion criteria included non-fluency in English, contraindication to MRI, pregnancy, and current incarceration/legal detention or placement on psychiatric hold.

**Figure 1:**
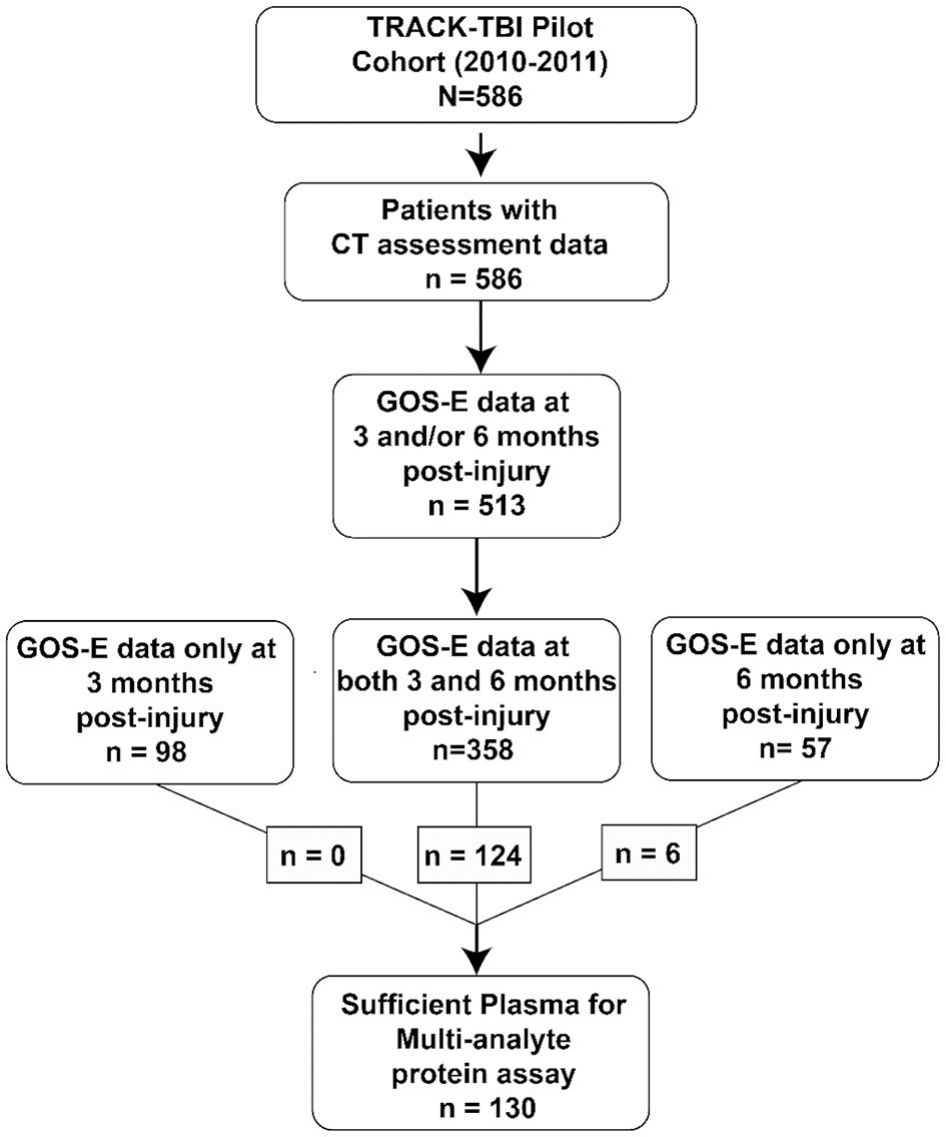
Patient selection. Flow diagram of the subset of patients from the TRACK-TBI Pilot dataset that was reused in the current set of analyses.

The current dataset utilizes only a small number of variables that were drawn from the previously published data versions, including age, presence of brain pathology on CT scan (CT positive), proteomic blood biomarker panel used in Huie et al. (10), and the GOS-E (3 and 6 months, Figure 1). This new merged data version has been recorded as a branch in the TRACK-TBI GitHub in order to capture this specific subset of patients, raw variables, and derived variables in perpetuity. This unique dataset version is available upon request.

### Proteomics Panel

Blood samples were collected at <24 h of injury, and plasma was prepared following TBI Common Data Elements Biospecimens and Biomarkers Working Group guidelines. Samples were centrifuged, aliquoted, and frozen at −80°C for batch processing. Frozen plasma samples were analyzed by Myriad Rules-Based Medicine (Myriad RBM, Austin, TX) and quantified using a multiplexed fluorescent immunoassay profile (HumanMAP v2.0).

### CT Scan

A brain CT was performed on all patients within 24 h of their arrival in the emergency department (ED presentation). CT data collection and interpretation were performed in accordance with TBI-CDE Working Group (22), with a blinded board-certified neuroradiologist reviewing the de-identified, anonymized scans. Rotterdam and Marshall scores were also determined. A positive CT indicated any intracranial pathology as determined by the neuroradiologist.

### Outcome Measures

The GOS-E was used to assess overall functional disability at 3 and 6 months post-injury. For consistency with prior work (10, 23), GOS-E scores were dichotomized in two ways: good recovery (GOS-E > 4) vs. poor recovery (GOS-E ≤ 4) and complete recovery (GOS-E = 8) vs. incomplete recovery (GOS-E < 8). To determine if patients' recovery improved between 3 and 6 months post-injury, a difference score was calculated (GOS-E at 6 months – GOS-E at 3 months).

### Missing Values in Proteomics

Protein values were considered missing if they fell outside the range of detection. A threshold for missingness for any biomarker was then set so that if any particular protein was outside the limit of detection for more than 50 patients (i.e., 30% missingness), then that protein would be excluded from analysis. This thresholding resulted in 15 biomarkers removed from analysis due to missingness. Fifty-seven biomarkers were subsequently included for analysis. For those remaining proteins, we then applied a constant-value imputation approach: for protein levels that were below the limit of quantification (LOQ), a value lower LOQ × 0.5 was imputed using the LOQ of the respective protein. For those above the LOQ, the upper LOQ value was used. By removing high-missingness variables prior to imputation with constant values, we reduce the likelihood of misrepresenting the relationship between the measured proteins and injury outcome that were the result from technical limitations of the assay.

### Statistics and Data Visualization

All analyses were performed using the open-source programming language R (24) (version 3.6.3). The *tidyverse* (25) R package was utilized for dataframe manipulation and data wrangling. For general data visualization and 3d surface plots, the *ggplot* (26) and *plotly* (27) R packages were utilized. Missing data analysis and visualization were generated using the *naniar* (28) R package. After biomarkers that exceeded the threshold of missingness were removed, the protein biomarker data were corrected for batch effects by within-batch *z*-score standardization. Using the “princals” function from the *Gifi* (29) R package, we then performed non-linear principal component analysis (NL-PCA) on the batch-corrected protein biomarker data to reduce high dimensionality. Specifically, NL-PCA was performed to allow for 2° splines and 57 total principal components (PCs) in the solution (matching the 57 biomarkers used as input to the NL-PCA). Permutation tests were then run using the *syndRomics* (30) R package to identify the PCs with variance accounted for (VAF) and standardized loadings significantly above chance (adjusted *p*-value > 0.05). Bar maps were additionally generated using the *syndRomics* R package. Hypothesis testing was conducted using linear regression for continuous outcome variables or logistic regression for dichotomized outcome variables, and Welch's unequal variance *t*-test for two group differences. Regression models were produced using the “lm” and “glm” functions from the *stats* (24) R package with additional type-III analysis of variance (ANOVA) performed to derive significance of main effects and interactions using the *car* (31) R package. To determine the confidence interval of the marginal effect of P2 on the change in GOS-E outcome between 3 and 6 months post-injury, we utilized the “deltamethod” function from the *msm* (32) R package.

## Results

### Age Correlates With Worse GOS-E Outcome and CT-Positive TBI

**Figure 2:**
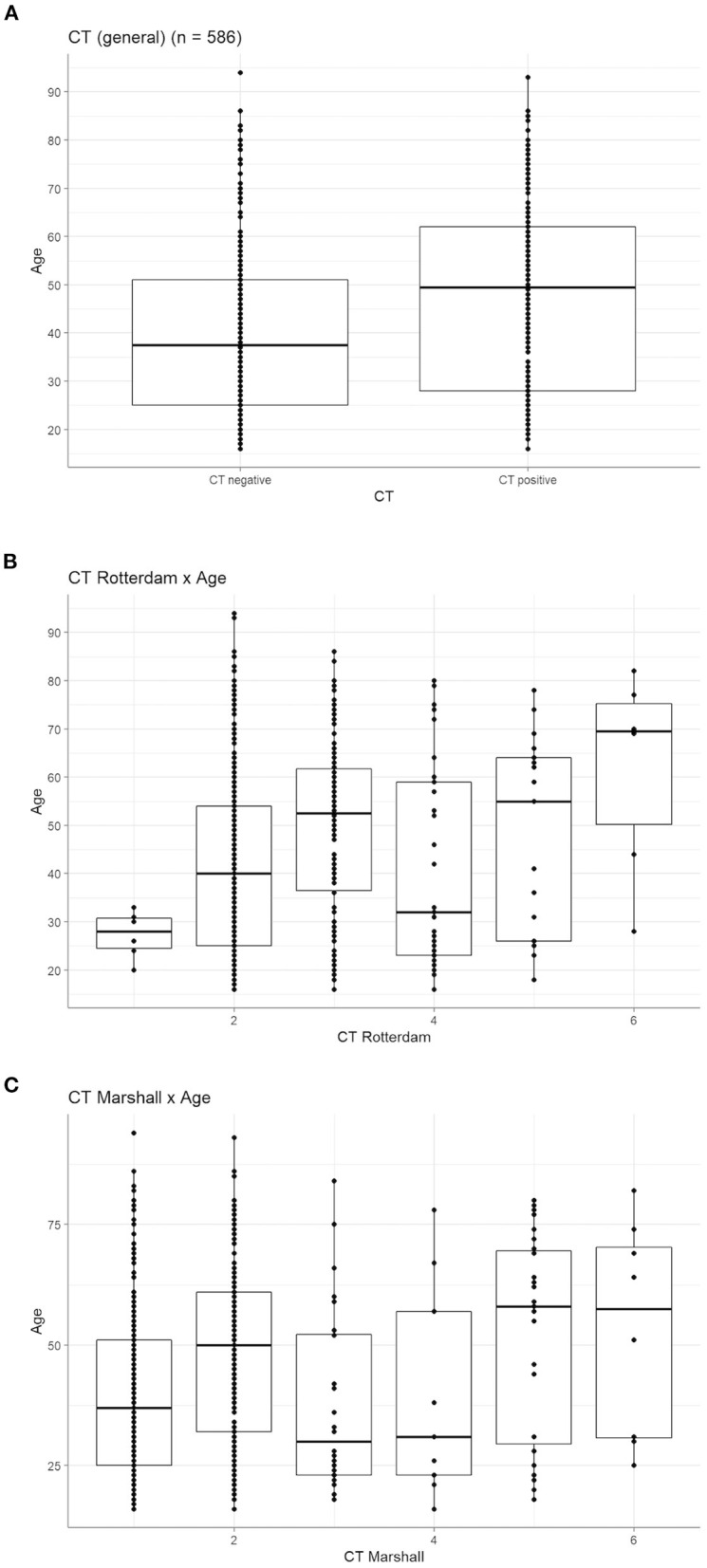
Age correlates with worse CT pathology. **(A)** Age is significantly higher in those with a positive diagnosis of CT pathology (Welch's *t* = 5.61, *p* < 0.0). **(B)** Age significantly differed across the six levels of the Rotterdam CT score [*F*_(1,584)_ = 17.34, *p* < 0.001] and the **(C)** six levels of the Marshall CT score [*F*_(1,584)_ = 18.25, *p* < 0.001]. For both scores, the two highest scores (indicative of more severe injury) have a higher median age than all other CT scores.

The first aim was to determine whether age predicts CT pathology and/or outcome on GOS-E at 3 and 6 months post-injury. Results showed that the patient population with positive CTs was significantly older than those who were CT negative (Figure 2A, Welch's *t* = 5.61, *p* < 0.01). Rotterdam scores also differed significantly by age [Figure 2B, *F*_(1,584)_ = 17.34, *p* < 0.001] as did Marshall scores [Figure 2C, *F*_(1,584)_ = 18.25, *p* < 0.001]: patients with higher scores (i.e., more severe injury by CT) on both measures tended to be older.

**Figure 3:**
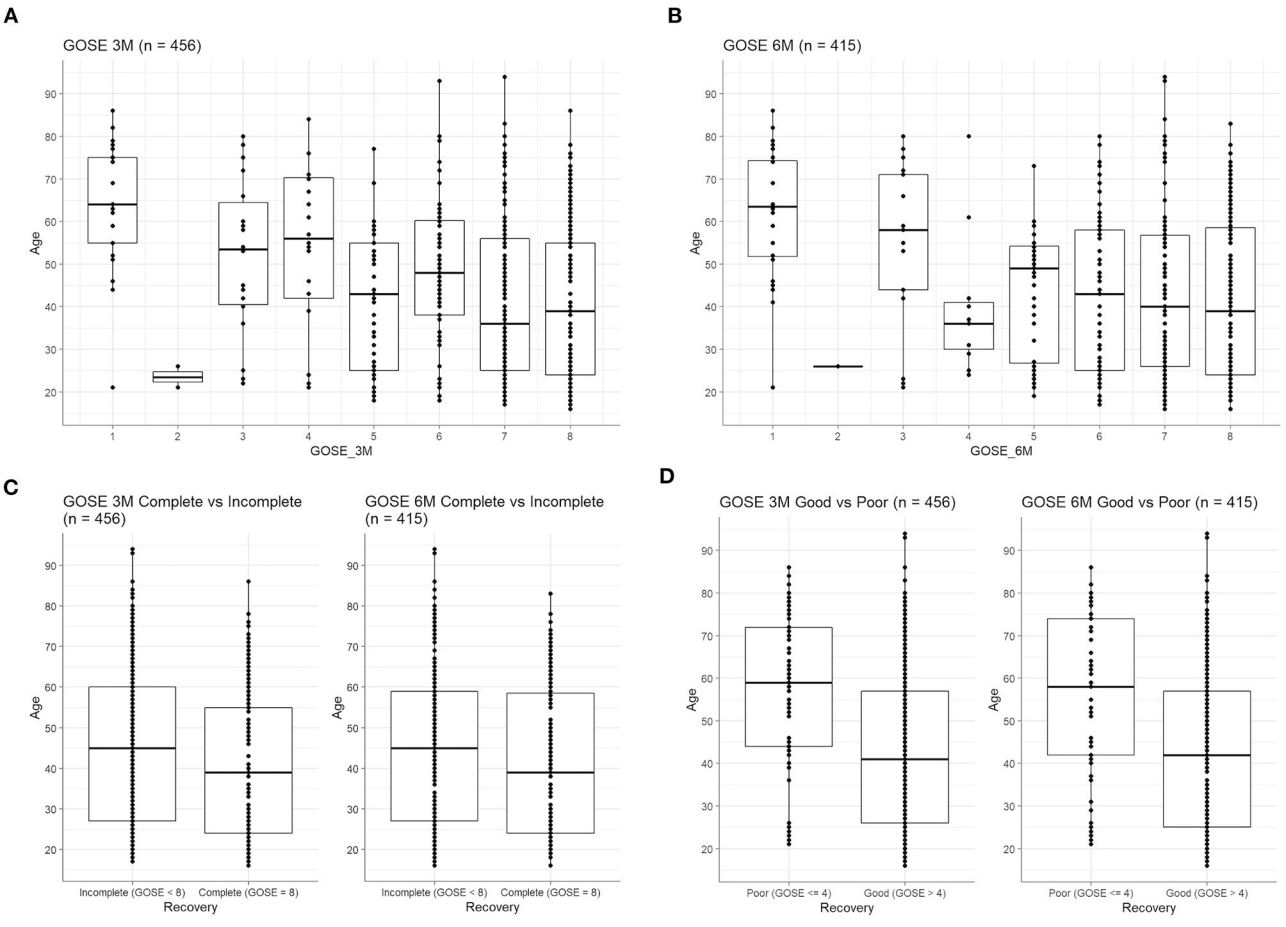
Age correlates with worse outcome on GOS-E. Age differs significantly across the eight levels of the GOS-E at **(A)** 3 months [*F*_(7,448)_ = 8.0, *p* < 0.001] and **(B)** 6 months [*F*_(7,407)_ = 5.66, *p* < 0.001]. **(C)** GOS-E was dichotomized as incomplete recovery (GOS-E score < 8) vs. complete recovery (GOS-E score of 8). Patients with complete recovery were significantly younger, at 3 months (Welch's *t* = −2.34, *p* = 0.02), but not at 6 months (Welch's *t* = −1.77, *p* = 0.08). **(D)** GOS-E was dichotomized as poor recovery (GOS-E score of 4 or below) vs. good recovery (GOS-E score of >4). Patients with poor recovery were significantly older, at both 3 months (Welch's *t* = −5.54, *p* < 0.001) and 6 months (Welch's *t* = −4.80, *p* < 0.001).

Worse functional outcome as captured by lower GOS-E scores was also correlated with older patient age at both 3 months [Figure 3A, *F*_(7,448)_ = 8.0, *p* < 0.001] and 6 months post-injury [Figure 3B, *F*_(7,407)_ = 5.66, *p* < 0.001]. Similarly, patients with complete recovery at 3 months were significantly younger (Figure 3C, Welch's *t* = −2.34, *p* = 0.02), but not at 6 months (Figure 3C, Welch's *t* = −1.77, *p* = 0.08). When GOS-E was dichotomized as poor recovery (≤ 4) or good recovery (>4), those with good recovery were also significantly younger (Figure 3D) at both 3 months (Welch's *t* = −5.54, *p* < 0.001) and 6 months (Welch's *t* = −4.80, *p* < 0.001).

### TBI Produces Multivariate Inflammatory Profile

**Figure 4:**
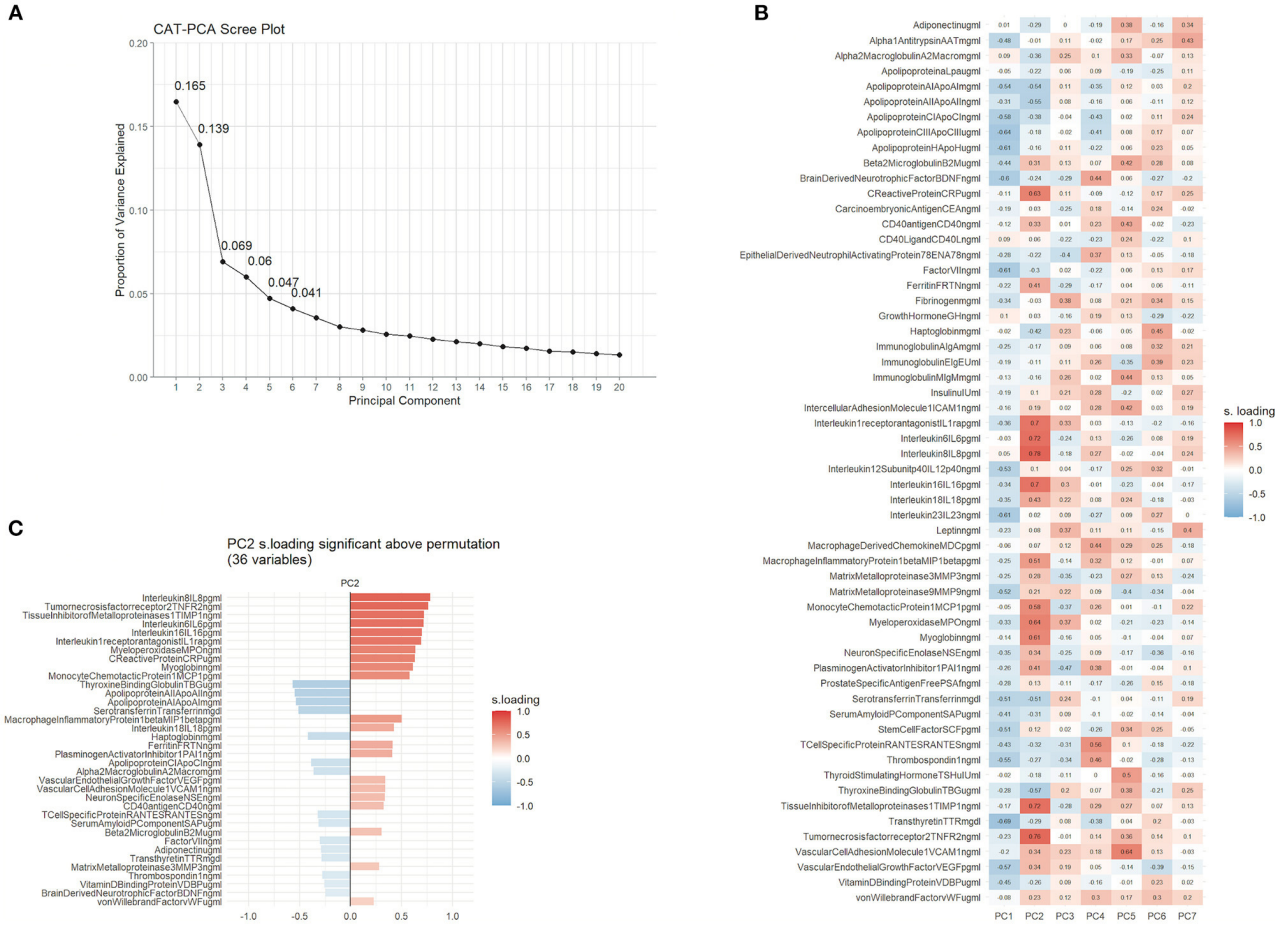
TBI produces multivariate inflammatory profiles. Non-linear PCA of multianalyte assay of 58 proteins. **(A)** Scree plot showing the proportion of overall variance that was accounted for by each orthogonal PC. The first component (PC1) accounted for 16.5% of the variance, followed by 13.9% of variance accounted for by PC2. There was a precipitous drop in variance accounted in PCs after PC2. **(B)** Matrix of the first seven PCs by 58 proteins. Heat map shows higher PC loadings in red and lower PC loadings in blue. A biologically interpretable pattern of loadings was identified in PC2, with proteins that loaded strongly being predominantly associated inflammation. **(C)** Permutation testing in PC2 indicated that the loadings for 36 of the 58 proteins were significantly above chance.

In order to reuse TRACK-TBI Pilot data to test whether age impacts proteomic expression, we first sought to replicate and improve upon prior multivariate proteomic analyses from this dataset (10). While the prior analysis used a linear PCA with optimal scaling, the current model used a non-linear approach as well as handled missing data above and below the limit of quantification differently than the prior published work (see Methods). Here, a permutation test for significant PCs was additionally used to objectively determine the number of significant principal components. This permutation test of VAF revealed that the first six PCs accounted for variance above chance. A second permutation test of significant loadings found that 33 proteins in PC1 and 35 proteins in PC2 loaded strongly enough to be above chance (adjusted *p*-value < 0.05). Although the VAF permutation determined that six PCs accounted for variance above chance (Figure 4A), no further PCs after PC2 had a protein loading that was significant (Figure 4B), suggesting that further analysis and interpretation should be limited to PC1 and PC2 only. The first two PCs together accounted for 28.7% of the variance (16.4% variance accounted for in PC1 and 12.3% variance accounted for in PC2). PC2 revealed a biologically interpretable loading pattern that strongly resembled the pattern seen in Huie et al. (10). The proteins with the strongest positive loadings on PC2 included pro-inflammatory proteins IL-8, IL-6, TIMP1, and C-reactive protein, while those that loaded in the opposite direction included anti-inflammatory or protective proteins including apolipoproteins AI, AII, and CI, as well as BDNF and serotransferrin (Figure 4C). Taken as a whole, the pattern of strong loading proteins indicates a broadly inflammatory profile, as seen in the previously published PCA of the same protein assay (10).

### Inflammatory Biomarker Profile Is Associated With Poor Outcome and CT-Positive TBI

**Figure 5:**
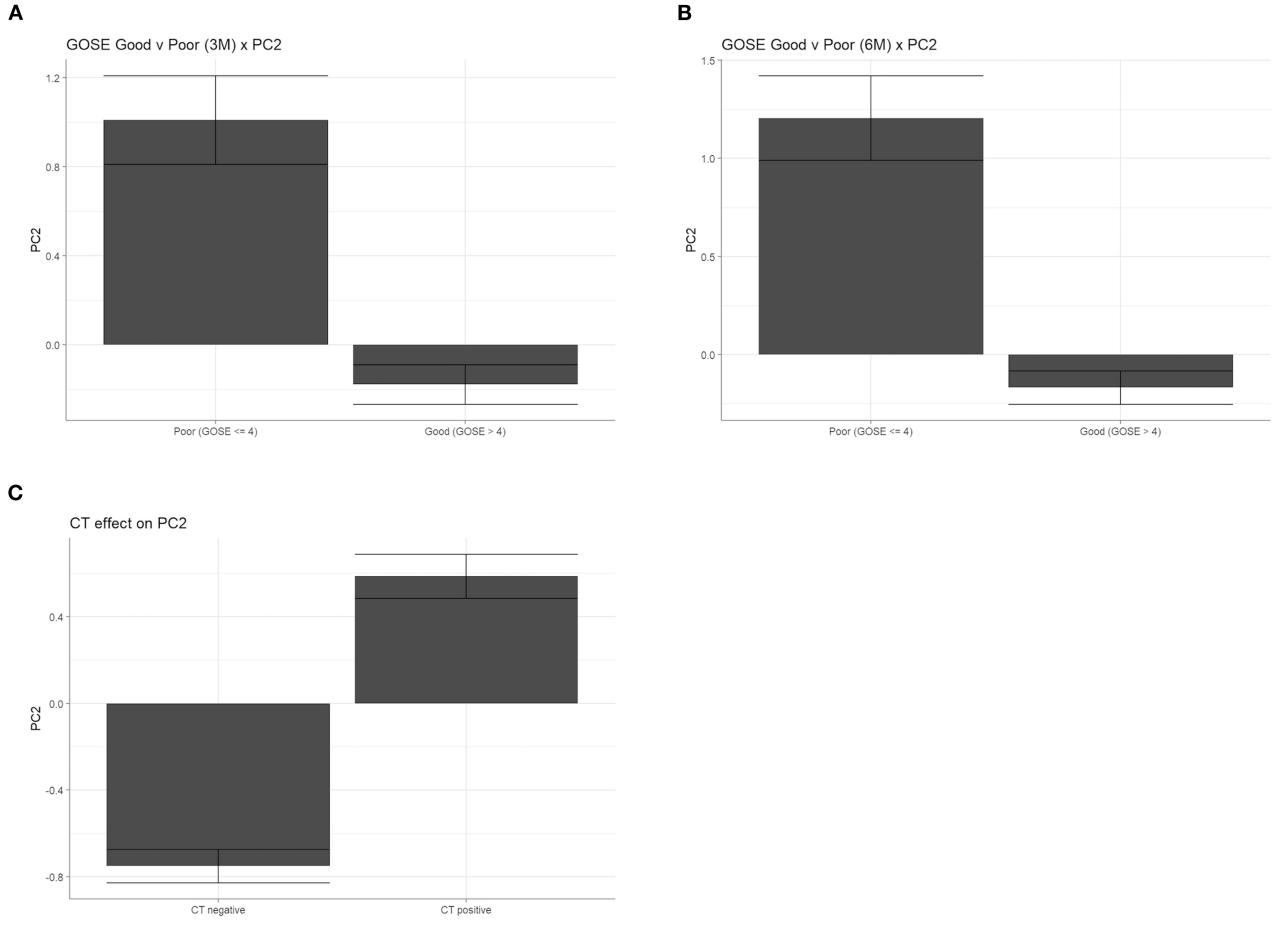
The inflammatory biomarker profile is associated with poor outcome and CT pathology. Individual PC scores for each patient that were generated from the inflammatory second PC, in which higher values are indicative of higher pro-inflammatory expression. Inflammatory PC scores were significantly higher in patients with poor recovery (GOS-E ≤ 4) vs. good (GOS-E > 4) recovery, at both 3 months (**A**, Welch's *t* = −5.43, *p* < 0.01) and 6 months (**B**, Welch's *t* = −5.46, *p* < 0.01). **(C)** Patients with positive diagnosis on CT pathology had significantly higher PC2 inflammatory scores (Welch's *t* = 10.53, *p* < 0.01).

The inflammatory biomarker profile revealed by proteomic PC2 is represented by PC scores for each patient, with higher scores indicating a more inflammatory proteomic composition and lower scores indicating an anti-inflammatory composition. Using this score as an injury response measure, we then determined whether patients with different outcomes expressed different inflammatory biomarker values. Results showed that patients with poor (GOS-E ≤ 4) recovery had significantly higher inflammatory PC scores than those with good (GOS-E > 4) recovery at both 3 months (Figure 5A, Welch's *t* = −5.43, *p* < 0.01) and 6 months (Figure 5B, Welch's *t* = −5.46, *p* < 0.01). Similarly, patients with CT-positive TBI had significantly higher inflammatory PC scores than those who were CT negative (Figure 5C, Welch's *t* = 10.53, *p* < 0.01). These findings replicate those seen in Huie et al. (10).

### Age Moderates the Effect of Inflammation on Outcome After TBI

**Figure 6:**
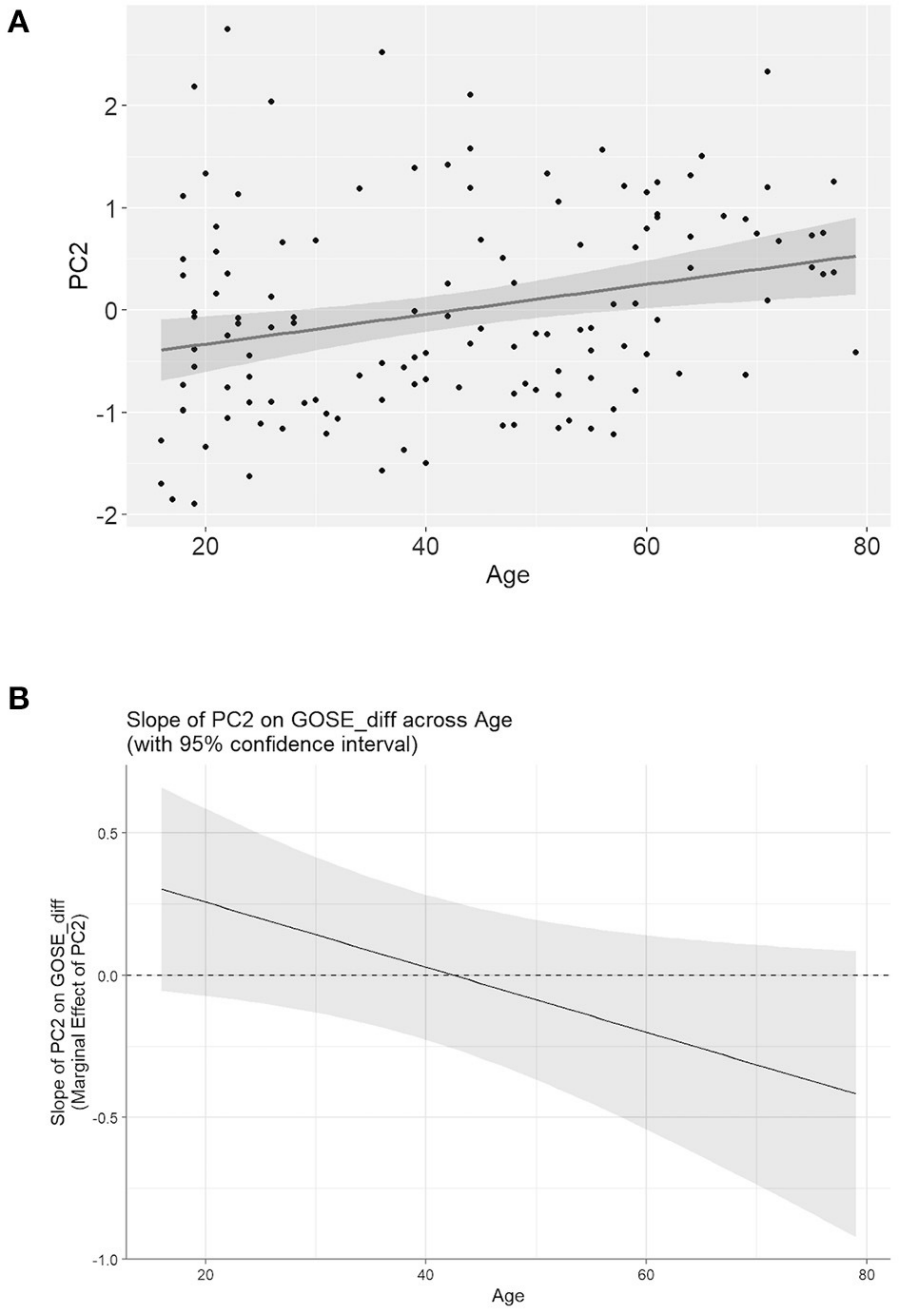
Age moderates the effect of inflammation on outcome after TBI. **(A)** Age significantly predicts inflammatory PC score (*R*^2^ = 0.062, *p* < 0.01), indicating greater multivariate inflammatory protein expression as age increases. **(B)** Visualization of the interaction between age and inflammation on improvement in GOS-E score from 3 to 6 months post-injury. The Y-axis represents the slope value of the effect of inflammatory PC2 score on change in GOS-E score. The negative relationship between slope value and age indicates that as age increases, the relationship between inflammation and improvement in recovery becomes more negative. This finding suggests a more detrimental relationship between inflammation and recovery for older TBI patients.

To determine whether the association between inflammation and outcome is affected by age, we first asked whether there is an overall association between age and the multivariate inflammatory biomarker panel. We found that age significantly correlates with inflammatory PC score, with a higher score (indicating a more pro-inflammatory profile) as patient age increases (Figure 6A, *R*^2^ = 0.062, *p* < 0.01). Next, the goal was to determine whether age interacts with the inflammatory PC score on GOS-E outcome. There was no significant interaction between age and inflammatory PC score for good/poor or complete/incomplete GOS-E recovery at either 3 or 6 months. Interestingly, there was a significant interaction between age and inflammatory PC score on the *change* in GOS-E between 3 and 6 months (age × PC score, LR chi-square = 5.31, *p* = 0.02) even after controlling for CT positivity. To better understand the nature of this interaction, we explored how the effect of inflammation on GOS-E improvement might differ across age. Figure 6B shows a decrease in the slope of the relationship between inflammatory PC score and GOS-E improvement as age increases. This finding suggests that in younger patients, an improvement in GOS-E is associated with increased inflammation; conversely, increased inflammation in older patients is associated with less robust recovery between 3 and 6 months post-injury.

## Discussion

This work aimed to illustrate the utility of data reuse for insight discovery. The governing principles of data management and stewardship are based on data being FAIR: findable, accessible, interoperable, and reusable. By harmonizing subsets of data from previously published work, there is an opportunity to test new hypotheses, improve on prior techniques, and gain knowledge that may otherwise be missed. The reuse of data in new ways also accelerates the process of discovery, allowing for novel recombination of data that maximizes the return on the costly investment of data collection and curation. The reuse and recombination of shared data will ultimately bring light to the “long tail of dark data” (5, 10).

The results of this data reuse reproduced and expanded on prior work showing that recovery after TBI is often age dependent. We found that patients who exhibited complete recovery (GOS-E = 8) at 3 and 6 months after TBI were significantly younger than those with incomplete recovery. Similarly, those with a GOS-E of 4 and below were significantly older than those with a GOS-E > 4. Of course, the likelihood of confounding comorbidities is greater as age increases, and older age in and of itself may not be a causal factor. We and others have previously shown that even when controlling for possible confounders, poor outcome becomes more likely with age. Gardner et al. showed that for those over 55, moderate to severe TBI significantly increases the risk of developing dementia, and for those over 65, even mild TBI increased this risk. This result was seen despite adjustments for common predictors and compared the TBI patients to non-TBI trauma controls, rather than the general population, to account for the possibility of reverse causality (e.g., early stage dementia leading to TBI in the first place) (15). Similarly, de la Plata et al. found that greater decline (as measured by the Disability Rating Scale) was seen in older TBI patients compared to younger TBI patients (14). Importantly, they noted that although age was a significant factor in decline after TBI, a relatively small proportion of the variance in the Disability Rating Scale was explained by age alone and that other factors and comorbidities may have a greater influence. Nevertheless, a large body of work has made it clear that age is an important factor that must be accounted for to understand the trajectory of recovery after TBI, especially since the elderly make up the highest proportion of TBI-related hospitalizations (33).

Given the prominent roles of both age and inflammation on TBI prognosis, we sought to understand how these important factors may interact to affect recovery. We found that even when controlling for CT positivity, there remained a significant interaction between age and the multivariate inflammatory profile on improvement in GOS-E over time. Interestingly, this interaction appears to be characterized by a decrease in the value of the slope of the relationship between inflammation and GOS-E improvement across ages (Figure 6B). This finding suggests that for younger TBI patients, an increase in inflammation is associated with *greater* functional improvement, while in older patients, increased inflammation tends to predict less improvement. Previous work supports the notion that the effect of inflammation on recovery may be age dependent. Pre-clinical study of age and inflammation found that the infiltration of inflammatory macrophages to the central nervous system (CNS) after experimental TBI was significantly increased in older animals relative to younger animals (34). Similarly, we found that in a TBI aging model, age-dependent inflammation was a significant contributor to cognitive impairment (17). Recent work using deep machine learning has derived an algorithm that uses inflammatory biomarkers to determine one's “inflammatory age,” based on the idea that the increase in chronic inflammation is an inevitable consequence of aging (35). This concept is related to the notion of “immunosenescence,” the dysregulation of immune function due to aging (36, 37). From this perspective, the immune challenge presented by a TBI in aging patients may work to undermine recovery by inducing detrimental inflammation that is not as easily resolved as in younger patients.

It is important to note that the interaction between age and inflammation on recovery found here was highly variable, as seen in the wide confidence intervals in Figure 5B. Thus, it is likely that a number of other confounders may account for the large amount of variance observed. For instance, given that the interaction of inflammation (measured within 24 h of injury) with age appears to have an affect on the course of recovery from 3 to 6 months, another source of variance may be the regulation of the inflammatory proteomic response over time.

Future studies will be needed to determine the static and dynamic mitigating factors that are likely at work. Similarly, as with many clinical datasets, generalization may be limited by blind spots of underrepresented groups among those enrolled, including those patients who did not warrant ED assessment at a high-level trauma center. It is also worth noting that although the unsupervised, data-driven approach of non-linear PCA revealed a predominantly inflammatory “identity” on PC2, the limitations of the assay were such that other proteins that would typically be candidate biomarkers for inflammation were found to be among the 15 proteins that were outside the level of detection, including TNF alpha, IL1-B, and macrophage inflammatory protein alpha. Thus, future work that is meant to specifically target the role of inflammation will require a more hypothesis-driven approach, with the *a priori* selection of biomarkers specific to inflammation. This kind of limitation may be typical for data reuse in the future and should be recognized and addressed: in the repurposing of prior data, there exists a possibility that the data may not always be ideal for comprehensively addressing a scientific question. But as long as the methodology and results are rigorous and transparent, building upon such findings can be undertaken in good faith.

## Data Availability Statement

The data analyzed in this study is subject to the following licenses/restrictions: a subset of the data is publicly available at https://doi.org/10.1371/journal.pone.0169490.s011. Access to corresponding proteomics data will be granted upon approved request. Requests to access these datasets should be directed to https://tracktbi.ucsf.edu/sites/tracktbi.ucsf.edu/files/TRACK-TBI%20Collaboration%20Proposal%20Request%20Form_0.pdf.

## Ethics Statement

This study was carried out in accordance with the recommendations of the University of California San Francisco (UCSF) Institutional Review Board of record, the Committee on Human Research (CHR). All subjects gave written informed consent in accordance with the Declaration of Helsinki. The protocol was approved by the UCSF CHR #10-00011.

## Author Contributions

JH, AC, AT-E, JN, and AF contributed to data curation and analysis. All authors contributed to interpretation of results, writing, and revising manuscript.

## Funding

This study was supported by National Institutes of Health Grants: R01NS088475 (AF); R01NS122888 (AF); UH3NS106899 (AF); and U24NS122732 (AF); Department of Veterans Affairs: 1I01RX002245 (AF) and I01RX002787 (AF); and Wings for Life Foundation and Craig H. Neilsen Foundation (AF), NIH/NINDS F32NS117728 (AC).

## Conflict of Interest

The authors declare that the research was conducted in the absence of any commercial or financial relationships that could be construed as a potential conflict of interest.

## Publisher's Note

All claims expressed in this article are solely those of the authors and do not necessarily represent those of their affiliated organizations, or those of the publisher, the editors and the reviewers. Any product that may be evaluated in this article, or claim that may be made by its manufacturer, is not guaranteed or endorsed by the publisher.
